# COVID-19 vaccine waning and effectiveness and side-effects of boosters: a prospective community study from the ZOE COVID Study

**DOI:** 10.1016/S1473-3099(22)00146-3

**Published:** 2022-07

**Authors:** Cristina Menni, Anna May, Lorenzo Polidori, Panayiotis Louca, Jonathan Wolf, Joan Capdevila, Christina Hu, Sebastien Ourselin, Claire J Steves, Ana M Valdes, Tim D Spector

**Affiliations:** a Department of Twin Research and Genetic Epidemiology, King's College London, London, UK; b School of Biomedical Engineering & Imaging Sciences, King's College London, London, UK; c Zoe Limited, London, UK; d Nottingham NIHR Biomedical Research Centre at the School of Medicine, University of Nottingham, Nottingham, UK

## Abstract

**Background:**

With the surge of new SARS-CoV-2 variants, countries have begun offering COVID-19 vaccine booster doses to high-risk groups and, more recently, to the adult population in general. However, uncertainty remains over how long primary vaccination series remain effective, the ideal timing for booster doses, and the safety of heterologous booster regimens. We aimed to investigate COVID-19 primary vaccine series effectiveness and its waning, and the safety and effectiveness of booster doses, in a UK community setting.

**Methods:**

We used SARS-CoV-2 positivity rates in individuals from a longitudinal, prospective, community-based study (ZOE COVID Study), in which data were self-reported through an app, to assess the effectiveness of three COVID-19 vaccines (ChAdOx1 nCov19 [Oxford-AstraZeneca], BNT162b2 [Pfizer-BioNtech], and mRNA1273 [Moderna]) against infection in the 8 months after completion of primary vaccination series. In individuals receiving boosters, we investigated vaccine effectiveness and reactogenicity, by assessing 16 self-reported systemic and localised side-effects. We used multivariate Poisson regression models adjusting for confounders to estimate vaccine effectiveness.

**Findings:**

We included 620 793 participants who received two vaccine doses (204 731 [33·0%] received BNT162b2, 405 239 [65·3%] received ChAdOx1 nCoV-19, and 10 823 [1·7%] received mRNA-1273) and subsequently had a SARS-CoV-2 test result between May 23 (chosen to exclude the period of alpha [B.1.1.7] variant dominance) and Nov 23, 2021. 62 172 (10·0%) vaccinated individuals tested positive for SARS-CoV-2 and were compared with 40 345 unvaccinated controls (6726 [16·7%] of whom tested positive). Vaccine effectiveness waned after the second dose: at 5 months, BNT162b2 effectiveness was 82·1% (95% CI 81·3–82·9), ChAdOx1 nCoV-19 effectiveness was 75·7% (74·9–76·4), and mRNA-1273 effectiveness was 84·3% (81·2–86·9). Vaccine effectiveness decreased more among individuals aged 55 years or older and among those with comorbidities. 135 932 individuals aged 55 years or older received a booster (2123 [1·6%] of whom tested positive). Vaccine effectiveness for booster doses in 0–3 months after BNT162b2 primary vaccination was higher than 92·5%, and effectiveness for heterologous boosters after ChAdOx1 nCoV-19 was at least 88·8%. For the booster reactogenicity analysis, in 317 011 participants, the most common systemic symptom was fatigue (in 31 881 [10·1%] participants) and the most common local symptom was tenderness (in 187 767 [59·2%]). Systemic side-effects were more common for heterologous schedules (32 632 [17·9%] of 182 374) than for homologous schedules (17 707 [13·2%] of 134 637; odds ratio 1·5, 95% CI 1·5–1·6, p<0·0001).

**Interpretation:**

After 5 months, vaccine effectiveness remained high among individuals younger than 55 years. Booster doses restore vaccine effectiveness. Adverse reactions after booster doses were similar to those after the second dose. Homologous booster schedules had fewer reported systemic side-effects than heterologous boosters.

**Funding:**

Wellcome Trust, ZOE, National Institute for Health Research, Chronic Disease Research Foundation, National Institutes of Health, Medical Research Council

## Introduction

The two-dose COVID-19 vaccination campaign substantially reduced hospitalisations and deaths despite high infection rates.^1^, ^2^, ^3^ However, the effectiveness against infection, as happens also for other vaccines, wanes within months of the second dose.^4^, ^5^ Studies in Qatar showed substantial waning^6^, ^7^ in effectiveness against SARS-CoV-2 infection from month 4 after the second dose for BNT162b2 (tozinameran; Pfizer-BioNtech),^6^, ^7^ although effectiveness against severe disease, hospitalisation, and death remained high 6 months after vaccination for both BNT162b2 and mRNA-1273 (elasomeran; Moderna).^6^, ^7^, ^8^

A systematic review of 39 studies showed vaccine effectiveness against symptomatic SARS-CoV-2 infection in the general population to be 89–97% for BNT162b2, 92% for ChAdOx1 nCoV-19 (Oxford-AstraZeneca), and 94% for mRNA-1273.^9^ However, vaccine effectiveness has been reported to drop to 44·1% with ChAdOx1 nCoV-19 or to 62·5% with BNT162b2 by week 20 after the second dose.^10^, ^11^ Risk of infection also increased considerably 6 months after vaccination in data from the National Israeli database^12^ and in a study of 780 225 individuals in the USA, with the increased risk being lower for mRNA vaccines (BNT162b2 and mRNA-123) than for Ad.26.COV2.S (Janssen), a viral vector vaccine.^13^ With the addition of novel COVID-19 variants of concern,^14^ several countries have offered COVID-19 vaccine boosters to the highest-risk groups to mitigate the pandemic.^15^ A booster dose of the BNT162b2 vaccine reduced the rates of both infection and severe COVID-19 illness in the Israeli population older than 60 years^16^ and overall.^17^ Boosters of mRNA-based vaccines were also safe and effective in randomised controlled trials,^18^, ^19^ with good immunogenicity observed for both homologous and heterologous booster doses.^18^ After receiving a booster dose, protection against symptomatic infection increased to over 93·1%,^10^, ^11^ resulting in a proposed regimen of universal boosters 6 months after the second dose.^20^, ^21^

**Research in context**

**Evidence before the study**

We searched PubMed for articles published up to Dec 20, 2021, using the terms “vaccine effectiveness waning” or “vaccine booster” and “COVID-19”. We found reviews summarising that titres of binding and neutralising antibodies wane over time for all vaccines and that this is also applicable to COVID-19 vaccines. For SARS-CoV-2, a preprint suggested that vaccine effectiveness was 44·1% for ChAdOx1 nCoV-19 (Oxford-AstraZeneca) and 62·5% for BNT162b2 (Pfizer-BioNtech) at least 20 weeks after receiving the second dose. Similar results have been reported in Qatar, but effectiveness against hospitalisation and death remained high after 6 months. Risk of infection has also been shown to increase considerably 6 months after vaccination in a large study in US veterans, with the increase in risk being much lower for mRNA-based vaccines than for Ad.26.COV2.S (Janssen), a viral vector-based vaccine. Two Israeli studies reported that a booster dose after vaccination with BNT162b2 could raise protection against symptomatic infection up to 93·1%. The COV-BOOST randomised controlled trial found that booster schedules increased both humoral and cellular responses to SARS-CoV-2, and that the side-effects were similar to those seen with primary vaccination.

There is a gap in knowledge regarding the actual waning in vaccine effectiveness against infection of both viral vector and mRNA COVID-19 vaccines after 5 months by demographic groups, and the restoration of effectiveness by boosters, particularly that of heterologous booster schedules, along with the side-effect profiles for homologous and heterologous boosters in the community.

**Added value of this study**

We report that both for mRNA (mRNA-1273 [Moderna] and BNT162b2) and viral vector (ChAdOx1 nCov-19) COVID-19 vaccines, effectiveness against infection substantially decreased over 5–8 months compared with 1 month after the second dose. Vaccine waning was lower among the younger age group (<55 years), with effectiveness above 76·7% 5 months after the second dose. We report no differences in effectiveness between months 5 and 6 for any of the vaccines. We also found that a booster dose at 6 months restored vaccine effectiveness to higher levels than those seen 1 month after the second dose. Systemic side-effects after booster vaccination were minor and affected 50 339 (15·9%) of 317 011 individuals, but post-vaccine systemic reactogenicity was higher in those receiving a heterologous booster schedule than in those receiving a homologous booster.

**Implications of all the available evidence**

The effectiveness against infection of COVID-19 vaccines waned considerably 5–8 months after primary vaccination, although it remained high, particularly among people younger than 55 years. Vaccine boosters were effective in restoring protection against infection and had a good safety profile in the community. The safety profile was better for homologous booster schedules than for heterologous ones.

The COV-BOOST multicentre, phase 2 randomised controlled trial in the UK (n=2878) found that booster schedules increased both neutralising antibodies and cellular responses within 28 days of administration.^18^ No safety concerns were raised, and side-effect profiles were similar to those seen with primary vaccination. As booster doses are offered to increasingly younger and less at-risk groups, assessing the number of months up to which vaccination is effective, and thus determining the ideal timing for boosters, becomes crucial for public health policy and resource optimisation. Moreover, issues regarding the safety of mix-and-match boosters are of considerable public concern, hence the need to compare systemic and localised side-effects for heterologous versus homologous boosters.

Here, we aimed to investigate vaccine effectiveness (of ChAdOx1 nCov19, BNT162b2, and mRNA1273) against infection in the 8 months following primary vaccination in a large prospective longitudinal community study of app users undergoing regular and ad-hoc SARS-CoV-2 testing. We further investigated the improved effectiveness and reactogenicity of a booster dose in a subset of individuals who had received one by Nov 23, 2021.

## Methods

### Study design and data source

This prospective cohort study analysed data acquired from UK voluntary participants in the ZOE COVID Study,^22^ who self-reported data through an app (appendix p 2). We analysed data collected from May 23, 2021, (to exclude the period of alpha [B.1.1.7] variant dominance) to Nov 23, 2021, when there was a data freeze. A consort diagram with the study design is presented in the appendix (p 6). Additional details for data sources, analyses, and selection of covariates are also provided in the appendix (pp 2–5).

Upon registration to the ZOE app, participants provide consent for their data to be used in COVID-19 research. They self-report demographic characteristics including age, sex, body-mass index (BMI), smoking, race or ethnicity, health-care worker status, and comorbidity data (appendix p 2). Participants are prompted to report any symptoms, SARS-CoV-2 tests and results, vaccination and booster details, and health-care access daily through app notifications.^23^ Individuals without symptoms are similarly encouraged to report through the app daily. Participants were asked if they had been vaccinated for COVID-19 and if so, to record the type of vaccine and date of administration. For 8 days from each vaccination day, users were asked daily whether they had any systemic or local side-effects, as previously described.^23^ Test positivity (regular or ad hoc) was self-reported through the app. The ZOE COVID Study app sends invites for testing to people reporting symptoms (including symptoms not recognised at a given timepoint by the UK Government as indicative of SARS-CoV-2 infection). When people reported more than one PCR or lateral flow result after vaccination, we selected the first test if positive or the latest test if all were negative.

Ethical approval for use of the ZOE app for research purposes in the UK was obtained from King's College London Ethics Committee (review reference LRS-19/20–18210), and all users provided consent for non-commercial use.

### Outcomes

Our primary outcome was infection rates (eg, self-reported lateral flow or PCR test positivity) in individuals 5–8 months after receiving both primary doses of the available vaccines and after receiving a booster shot with either BNT162b2 or mRNA-1273. Our secondary outcome was self-reported reactogenicity within 8 days of the booster dose.

As a sub-analysis, we also investigated illness severity, defined as having two of three respiratory symptoms (chest pain, persistent cough, and shortness of breath),^24^ and hospital admission in individuals testing positive for SARS-CoV-2 5–6 months after receiving both primary doses of the available vaccines.

### Statistical analysis

Statistical analysis was done with use of Python, version 3.7 (pandas, NumPy, and SciPy).

In participants vaccinated with two doses of BNT162b2, ChAdOx1 nCoV-19, or mRNA-1273 who were subsequently tested for SARS-CoV-2 infection, we investigated changes in infection rates in the 8 months after the second dose, compared with those of unvaccinated app users.^23^ After adjusting for age (<55 years and ≥55 years), sex, previous infection (binary variable), health-care worker status (binary variable), comorbidities (binary variable, with or without comorbidities), number of tests, and weekly incidence per million individuals in the UK at the time of the infection to control for background positivity level as previously described,^23^ we defined vaccine effectiveness, VE, as the following: VE = 1 – *RR*_i,n_ where the risk ratio RR is the exponential of the treatment coefficient in the Poisson regression model, *i*ε[BNT162b1, ChAdOx1 nCoV-19, mRNA-1273] and *n*ε[1, 2, 3, 4, 5, 6, 7, 8]. Test results of individuals who received a booster were excluded after their booster date.

Additionally, we tested the role of covariates in risk of infection post-vaccination by running stratified Poisson models (adjusted for confounders) on categories of age and comorbidities (appendix p 2). We then did sensitivity analyses in individuals who test frequently (ie, health-care workers), those who were previously infected, and those with symptomatic infection to ensure these were not a source of bias. We further assessed whether loss to follow-up was a source of bias, by comparing the baseline characteristics of individuals who stayed enrolled in the study and reported testing results several months post-vaccination with those of individuals who were lost to follow-up. We further investigated vaccine effectiveness against hospitalisation by running the same model, with hospitalisation as the endpoint.

We investigated the effectiveness of vaccine boosters in preventing infection in a subset of app users who received two primary doses of BNT162b2 or ChAdOx1 nCoV-19, received either a BNT162b2 or an mRNA-1273 booster dose between Sept 16 and Nov 22, 2021, and were aged 55 years or older. As a control group, we selected individuals aged 55 years or older who received two primary doses of BNT162b2 or ChAdOx1 nCoV-19 but had not yet taken up their booster dose. We used adjusted Poisson regressions to compare the positivity rates in individuals with booster doses versus those with only two doses. We obtained the estimate of the log difference in the positivity rates of individuals who received a booster and control individuals who received two vaccine doses from the Poisson regression model. We combined the estimated difference between these two groups to the estimated risk reduction compared with unvaccinated individuals (measured at 0–3 months post-vaccination; more details in appendix p 3).

To investigate systemic and local adverse effects in individuals after receiving a booster, we computed the percentage of users reporting side-effects in the 8 days following the injection. We also considered the symptomatology of the same people in the 8 days following their second dose to compare reactogenicity of third doses with that of second doses. We compared the reactogenicity of different vaccines using Pearl's adjustment (appendix p 3).

### Role of the funding source

ZOE developed the app for data collection as a not-for-profit endeavour. ZOE received a grant from the UK Department of Health and Social Care to provide ongoing surveillance data. Employees of the funder were involved in most aspects of the study.

## Results

For the analysis of vaccine effectiveness of two doses, we included 620 793 UK app users who reported being fully vaccinated and subsequently tested for SARS-CoV-2 with an RT-PCR-based test or a lateral flow test between May 23 (once the SARS-CoV-2 delta [B.1.617.2] variant became predominant) and Nov 23, 2021, and 40 345 unvaccinated users who had a PCR or lateral flow test result in the same period (appendix p 6). 204 731 (33·0%) individuals received two doses of BNT162b2, 405 239 (65·3%) received two doses of ChAdOx1 nCoV-19, and 10 823 (1·7%) received two doses of mRNA-1273 (demographic characteristics are shown in the table). The study sample was predominantly female (409 065 [61·9%] of 661 138) and 137 939 (20·1%) were obese (mean BMI 26·61 kg/m^2^, SD 5·33). On average, fully vaccinated individuals completed their second dose 3·84 months (IQR 3–5) before the analysis.

**Table:** Descriptive characteristics of the study population, by type of vaccine used in the primary immunisation series

		**BNT162b2 (n=204 731)**	**ChAdOx1 nCoV-19 (n=405 239)**	**mRNA-1273 (n=10 823)**	**Unvaccinated (n=40 345)**
Sex					
	Female	134 032 (65·5%)	242 829 (59·9%)	6235 (57·6%)	25 969 (64·4%)
	Male	70 699 (34·5%)	162 410 (40·1%)	4588 (42·4%)	14 376 (35·6%)
Age, years		50·0 (13·9);52 (38–62)	54·8 (9·9);56 (48–63)	39·1 (8·3);39 (33–46)	37·7 (13·2);34 (27–47)
BMI, kg/m^2^		26·6 (5·6)	26·8 (5·3)	25·2 (4·6)	25·4 (5·3)
Health-care workers		27 110 (13·2%)	9522 (2·3%)	84 (0·7%)	1794 (4·4%)
Comorbidities		41 136 (20·1%)	66 471 (16·4%)	755 (7·0%)	3958 (9·8%)
Infection post-vaccination		16 037 (7·8%)	45 384 (11·2%)	751 (6·9%)	6726 (16·7%)*
	PCR confirmed	11 491 (71·7%)	32 082 (70·7%)	525 (69·9%)	4868 (72·4%)*
	LFT confirmed	4546 (28·3%)	13 302 (29·3%)	226 (30·1%)	1858 (27·6%)*
Infections with symptom assessment		15 320 (7·5%)	43 706 (10·8%)	739 (6·8%)	4962 (12·3%)
Symptomatic infections post-vaccination		13 682 (6·7%)	40 354 (10·0%)	646 (6·0%)	4575 (11·3%)*
Booster		98 008 (47·9%)†	120 525 (29·7%)‡	0	0

Data are n, n (%), mean (SD), or mean (SD); median (IQR). BMI=body-mass index. LFT=lateral flow test.

* Infections during the study period.

† Data indicate that of 204 731 individuals who received two doses of BNT162b2 in the primary immunisation series, 98 008 received a booster dose, including 91 692 who received BNT162b2 and 6316 who received mRNA-1273.

‡ Data indicate that of 405 239 individuals who received two doses of ChAdOx1 nCoV-19 in the primary immunisation series, 120 525 received a booster dose, including 102 780 who received BNT162b2 and 17 745 who received mRNA-1273.

**Figure 1:**
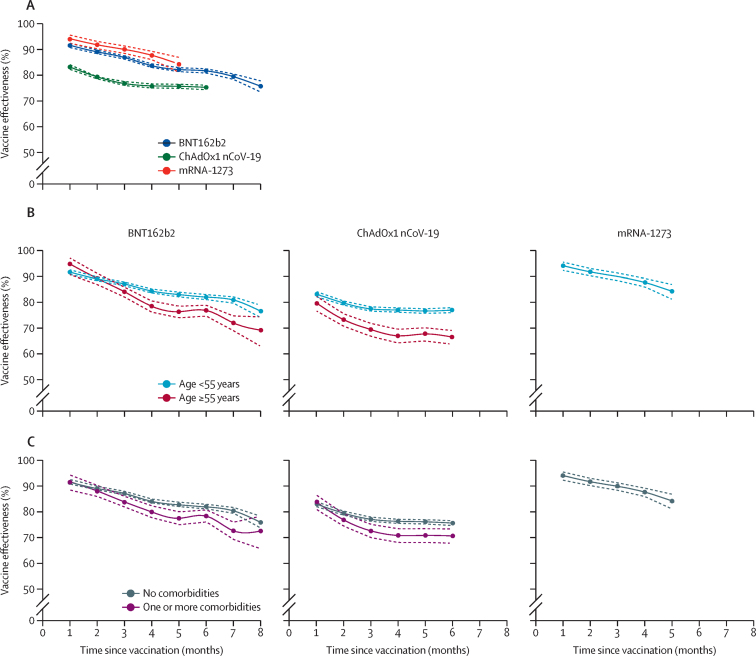
Primary immunisation series effectiveness against infection over time, overall (A) and by age (B) and presence of comorbidities (C) **w**The graphs represent the risk reduction for infection of the vaccinated group compared with the unvaccinated group by vaccine type and months since vaccination. Dotted lines indicate 95% CIs.

We investigated changes in infection rates after completing the second dose. After the second dose, 62 172 (10·0%) of 620 793 vaccinated individuals and 6726 (16·7%) of 40 345 unvaccinated controls tested positive for SARS-CoV-2 infection. Data were available for up to 8 months after the second dose for BNT162b2, for up to 6 months for ChAdOx1 nCoV-19, and for up to 5 months for mRNA-1273. In line with our previous reports,^23^ we observed that 1 month after the second dose, infection risk in the vaccinated group was significantly lower than in the unvaccinated population (vaccine effectiveness of 91·6%, 95% CI 90·7–92·4, for BNT162b2; 83·1%, 82·2–84·0, for ChAdOx1 nCoV-19; and 94·1%, 92·3–95·5, for mRNA-1273), after adjusting for confounders using Poisson regressions^25^ (figure 1, appendix pp 7–8). As depicted in figure 1A, vaccine effectiveness gradually started waning after the second shot. BNT162b2 effectiveness was 82·1% (81·3–82·9) at 5 months, 81·6% (80·8–82·4) at 6 months, and 75·7% (73·4–77·7) at 8 months; ChAdOx1 nCoV-19 effectiveness was 75·7% (74·9–76·4) at 5 months and 75·2% (74·3–76·1) at 6 months; and mRNA-1273 effectiveness was 84·3% (81·2–86·9) at 5 months (appendix pp 7–8).

For each vaccine, we observed a larger waning of effectiveness in individuals aged 55 years or older than in those younger than 55 years, with similar trends observed over time (figure 1B). For this analysis, we included 300 944 participants who were doubly vaccinated and younger than 55 years, of whom 41 137 (13·7%) tested positive for SARS-CoV-2, and 319 849 aged 55 years or older, of whom 21 035 (6·6%) tested positive. The control group consisted of unvaccinated participants: 34 355 younger than 55 years, of whom 5992 (17·4%) tested positive, and 5990 aged 55 years or older, of whom 734 (12·3%) tested positive.

At 5 months, BNT162b2 vaccine effectiveness was 76·3% (74·0–78·5) in those aged 55 years or older compared with 83·0% (82·0–83·8) in those younger than 55 years; at the same timepoint, ChAdOx1 nCoV-19 effectiveness was 67·8% (65·1–70·2) in those aged 55 years or older compared with 76·7% (75·9–77·6) in those younger than 55 years.

We found that individuals with comorbidities who received the BNT162b2 or ChAdOx1 nCoV-19 vaccine had lower vaccine effectiveness than individuals without comorbidities (eg, 77·5%, 74·9–79·9, *vs* 82·8%, 81·9–83·6, at 5 months with BNT162b2; and 70·8%, 68·0–73·5, *vs* 76·1%, 75·3–76·9, at 5 months with ChAdOx1 nCoV-19; figure 1C). For this analysis, 512 431 participants without comorbidities who were doubly vaccinated (52 058 [10·2%] tested positive) were compared with 36 387 unvaccinated individuals with no comorbidities (6106 [16·8%] tested positive); and 108 362 individuals with at least one comorbidity who were doubly vaccinated (10 114 [9·3%] tested positive) were compared with 3958 unvaccinated individuals with at least one comorbidity (620 [15·7%] tested positive). Because the mRNA-1273 vaccine was offered to younger individuals without comorbidities, we could not do analyses stratified by age or comorbidities.

We did sensitivity analyses in participants who test frequently (ie, health-care workers), those who were previously infected, and those with symptomatic infection; we found that vaccine effectiveness at 5 months was not substantially different in any of these subgroups compared with the main analysis (appendix p 9). To assess whether loss to follow-up was a source of bias, we compared the characteristics at baseline of individuals who stayed enrolled in the study and reported testing results several months post-vaccination with those of individuals who were lost to follow-up; we found that these groups were broadly similar (table; appendix p 10).

Vaccine effectiveness against severe infection and hospitalisation remained high 5–6 months after completion of the primary vaccination series (effectiveness against severe infection of 78·8%, 95% CI 77·1–80·3, and against hospitalisation of 84·1%, 81·0–86·7; appendix p 11). Moreover, vaccine effectiveness was higher among individuals younger than 55 years (effectiveness against severe infection of 79·2%, 77·4–80·8, and against hospitalisation of 84·3%, 80·7–87·2) than among individuals aged 55 years and older (effectiveness against severe infection of 66·5%, 57·5–73·5, and against hospitalisation of 80·4%, 70·7–86·9; appendix p 12). As the mRNA-1273 vaccine was offered to the younger age group with less severe infection outcomes, we could not do an analysis of effectiveness against severe illness or hospitalisation separately for this vaccine. For BNT162b2 and ChAdOx1 nCoV-19, vaccine effectiveness estimates were greater in younger than in older individuals (appendix p 12).

**Figure 2:**
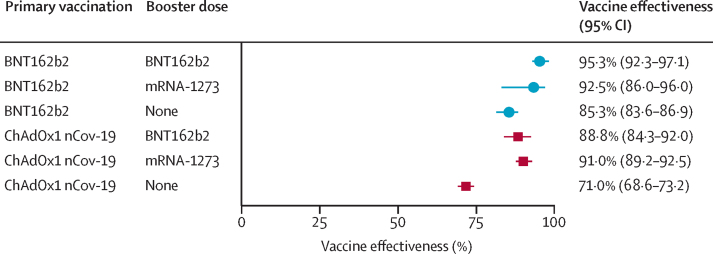
Effectiveness against infection of homologous and heterologous booster doses in individuals aged 55 years or older Error bars indicate 95% CI. Vaccine effectiveness estimates for booster doses (or two doses) in 0–3 months after immunisation compared with no vaccination are shown.

During the study period, 194 472 app users registered receiving booster shots with BNT162b2 and 24 061 with mRNA-1273. We assessed the effectiveness of homologous and heterologous booster doses in 135 932 participants aged 55 years or older who received a booster dose (2123 [1·6%] subsequently infected). For individuals who received a booster, we saw significant increases in effectiveness against infection in 0–3 months post-booster compared with the same time period after the second dose in 33 466 individuals aged 55 years or older doubly vaccinated without a booster (824 [2·5%] subsequently infected; appendix p 13). This translated to a vaccine effectiveness versus unvaccinated individuals aged 55 years or older of 95·3% (92·3–97·1) for homologous BNT162b2 schedules (n=63 632), 91·0% (89·2–92·5) for those receiving a BNT162b2 booster after two primary ChAdOx1 nCoV-19 doses (n=63 922), 88·8% (84·4–92·0) for those receiving an mRNA-1273 booster after two primary ChAdOx1 nCoV-19 doses (n=6000), and 92·5% (86·0–96·0) for those receiving an mRNA-1273 booster after two primary doses of BNT162b2 (n=2378; figure 2, appendix p 13).

We further investigated the occurrence of systemic and local adverse effects within 8 days after administration of the booster dose. 317 011 participants completed at least one daily report of systemic and local side-effects after receiving the booster (appendix pp 14–15). Of these, 27 761 (8·8%) received an mRNA-1273 third dose and 289 250 (91·2%) received BNT162b2; 134 637 (42·5%) participants received homologous prime–boost schedules and 182 374 (57·5%) received heterologous schedules. On average, the mean age of participants who received a booster was 65·4 years (SD 10·6) and the mean BMI was 26·5 kg/m^2^ (5·1).

**Figure 3:**
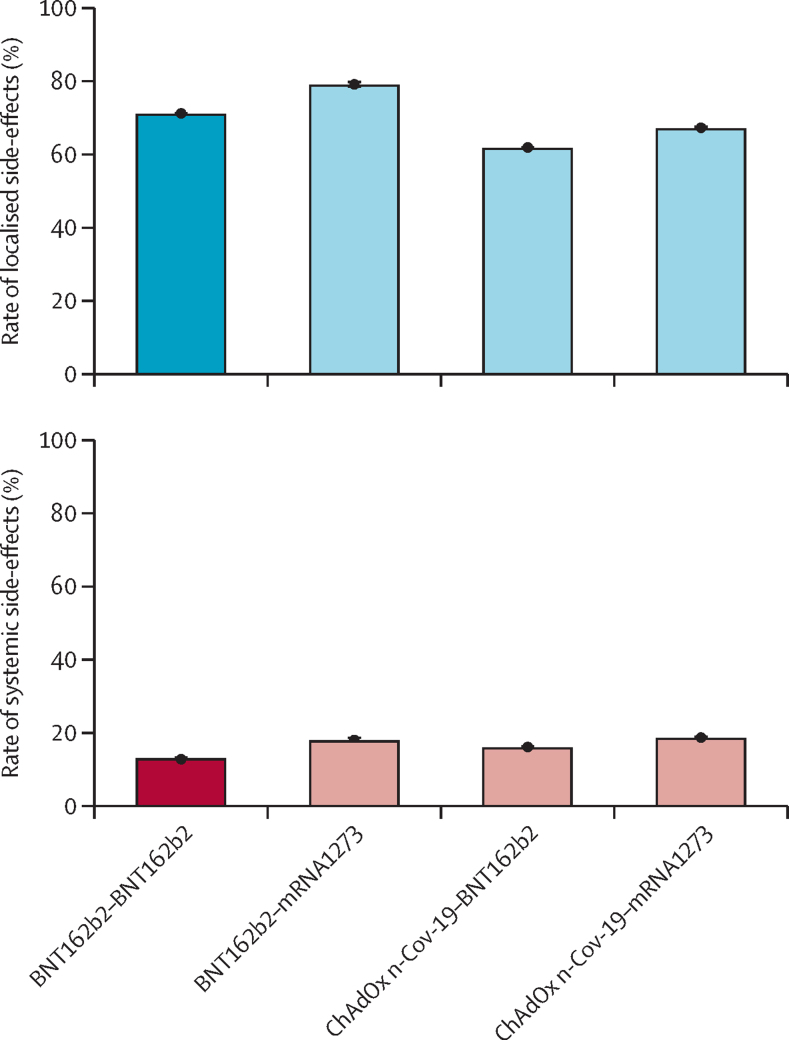
Proportion of participants self-reporting adverse effects to the ZOE COVID Study app within 8 days after receiving a booster Systemic and local side-effects after the booster are presented for homologous and heterologous dose combinations.

After the booster, 50 339 (15·9%) of 317 011 individuals reported having at least one systemic adverse effect and 232 596 (73·4%) reported one or more local effects within 8 days of the injection. The most commonly reported systemic side-effects were fatigue and headache, and the most frequently reported local side-effects were tenderness and pain around the site of injection (appendix p 14), the same as what was reported after the first two vaccine doses.^23^ For those receiving homologous BNT162b2 schedules, the proportion of participants who reported systemic side-effects after the booster was slightly lower than after the second dose (13·2%, 95% CI 13·0–13·3, for the third dose *vs* 19·2%, 19·0–19·4, for the second dose; odds ratio [OR] 1·6, 95% CI 1·5–1·6; p<0·0001) after adjusting for covariates (figure 3, appendix p 16).

Individuals who received a heterologous booster dose had higher rates of systemic adverse effects (17·9%, 17·7–18·1; 32 632 of 182 374) than those who received a homologous booster dose (13·2%; 17 707 of 134 637; OR 1·5, 1·5–1·6, p<0·0001, *vs* homologous BNT162b2). Among those on a heterologous schedule, participants receiving a third mRNA-1273 dose after a second BNT162b2 or ChadOx nCoV-19 dose were more likely to report systemic side-effects (18·0%, 95% CI 17·1–18·8) than those receiving the other heterologous combination (16·1%, 15·9–16·2, for ChadOx1 nCoV-19 followed by BNT162b2; OR 1·2, 1·2–1·3, p<0·0001).

Similarly, local side-effects after the third BNT162b2 dose were less frequent than those after the second dose (71·2%, 95% CI 71·0–71·5, after the third dose *vs* 76·6%, 76·0–76·8, after the second dose; OR 1·2, 1·2–1·3, p<0·0001). However, as before, people receiving heterologous booster doses reported on average more local side-effects than those receiving a homologous dose, with participants receiving a third mRNA-1273 booster after BNT162b2 more likely to report local side-effects than those receiving other heterologous combinations (figure 3; appendix p 16).

## Discussion

In this large-scale, community-based study of over 600 000 people, we found that, although there was substantial waning of COVID-19 vaccine effectiveness against infection 5–8 months after the second vaccine dose, effectiveness against infection remained high overall (above 75%) and particularly so among healthy individuals and those younger than 55 years (76·1% for all vaccine types). We also found that receiving a booster dose of BNT162b2 or mRNA-1273 6 months after the second primary dose restored vaccine effectiveness to higher levels than those seen 1 month after the second dose, for both BNT1622b2 and ChAdOx1 nCoV-19 primary immunisation series. Effectiveness against infection after boosters was higher than 88·8% for all heterologous and homologous schedules, supporting current policies in several countries,^15^ which encourage booster doses to reduce transmission of SARS-CoV-2. The values for effectiveness against infection seen in our study are consistent with those reported by a smaller UK-based study in individuals older than 50 years^10^ and by a large Israeli study.^16^

Waning of effectiveness against infection for COVID-19 vaccines that use novel technologies such as mRNA-based delivery is in line with what has been observed for more traditional vaccines such as the ones used for influenza, where the odds of testing positive for influenza increases by 16% per 28-day period between vaccination and testing.^26^ However, consistent with what is expected of vaccines that induce high T-cell responses,^27^ we found that ChAdOx1 nCoV-19 effectiveness against infection remained stable after 3 months, albeit lower than for BNT162b2, with no significant drop in effectiveness seen between months 3 and 6 in our data.

In addition to effectiveness against infection, our data show that 5–8 months after vaccination, individuals have additional protection against severe illness and hospitalisation, even if infected with SARS-CoV-2. For those aged 55 years or older, vaccine effectiveness against hospitalisation due to COVID-19 was over 80%, compared with that of individuals in the same age group who were unvaccinated and infected with SARS-CoV-2.

Our data show higher effectiveness against infection (measured as test positivity) after 5 months than those reported in other studies. For example, a study from Qatar^8^ reported complete waning against infection at 7 months or more after primary vaccination with BNT162b2, but the analysis included only 297 vaccinated people. Similar findings were reported among individuals older than 50 years in the UK.^10^ A much larger study^28^ from a health-care provider in the USA found 90% effectiveness of BNT162b2 after 5 months against hospitalisation but only 53% against infection. There are two crucial differences between these studies and ours. First, these studies assessed vaccine effectiveness including asymptomatic infections (which are more common than symptomatic ones), but our database contains primarily symptomatic infections and mostly fails to include this group. Second, our analyses are adjusted for previous infection. As time since vaccination increases, a higher proportion of the unvaccinated population becomes infected, hence the relative vaccine effectiveness against infection decreases regardless of actual waning, simply because of the increased levels of immunity in the control group. Therefore, it is probable that, by adjusting for this factor, our rates of vaccine effectiveness were higher than those of other studies.

The choice of a control sample is crucial, as highlighted by an Israeli study on vaccine effectiveness waning of BNT162b2.^8^, ^12^, ^28^ In that study, waning was assessed by comparing the likelihood of infection for the same individual at an earlier timepoint, because the majority of the Israeli population is vaccinated and the unvaccinated population is likely to be a biased sample. However, those vaccine effectiveness estimates assessed not merely waning, but also differences in vaccine effectiveness against changing dominant SARS-CoV-2 variants.

We report that systemic adverse effects, including headache and fatigue, affected 15·9% of participants after receiving the booster dose, and local effects affected 73·4% of participants. However, systemic side-effects were significantly higher after heterologous booster doses (affecting more than 17% of individuals) than after homologous booster schedules, which affected fewer than 12% of participants.

Our study has several strengths, among which are the large sample size, the fact that vaccine effectiveness for both the primary immunisation series and booster doses was assessed when infection pressure from the delta variant was the same, and reactogenicity reporting in a large sample of participants.

Our study also has some limitations. First, we used self-reported data, which can introduce information bias, including misclassification, or collider bias. Additionally, because of privacy concerns, we were unable to cross-reference participants' responses with national databases for infection or immunisation. We also assumed that all participants report symptoms in the same way. Second, participants using the app were a self-selected group and might not be fully representative of the general population. However, our app is able to produce estimates of population-level disease prevalence that agree with surveys with a representative design,^29^ suggesting behavioural issues are not substantially biasing our population. Moreover, we cannot rule out the presence of selection bias in who was tested after vaccination, as they might not be representative of the whole vaccinated population. Third, our measurements are limited by the booster rollout's focus on health-care workers, older age groups, and clinically vulnerable individuals, which was the UK Government's policy until Dec 1, 2021.^30^, ^31^ Finally, our study focused on the timescale of the predominant delta variant and might not be generalisable to other variants.

Overall, our data suggest that young, healthy adults 6 months after primary vaccination retain substantial immunity to SARS-CoV-2 variants dominant up until November, 2021. Our data indicate that booster doses are safe and effective, and systemic side-effects are less frequent for participants receiving homologous than heterologous doses.

## Data sharing

Anonymised research data are shared with third parties through the centre for Health Data Research UK (https://www.HDRUK.ac.uk). US investigators are encouraged to coordinate data requests through the COPE Consortium (https://www.monganinstitute.org/cope-consortium). Data updates can be found on https://covid.joinzoe.com.

## Declaration of interests

TDS, AMV, CJS, and SO are consultants to ZOE. JW, AM, LP, and JC are employees of ZOE. All other authors declare no competing interests.
