# Supplementary material for: COVID-19 vaccine waning and effectiveness and side-effects of boosters: a prospective community study from the ZOE COVID Study
Source: Lancet Infect Dis. 2022 Jul;22(7):1002–10. doi: 10.1016/S1473-3099(22)00146-3 (PMC8993156; doi:10.1016/S1473-3099(22)00146-3)
Supplement: Supplementary appendix [file mmc1.pdf]

THE LANCET
Infectious Diseases

Supplementary appendix

This appendix formed part of the original submission and has been peer reviewed. We post it as supplied by the authors.

Supplement to: Menni C, May A, Polidori L, et al. COVID-19 vaccine waning and effectiveness and side-effects of boosters: a prospective community study from the ZOE COVID Study. *Lancet Infect Dis* 2022; published online April 8. https://doi.org/10.1016/S1473-3099(22)00146-3.

COVID-19 vaccine waning and effectiveness and side effects of boosters: a prospective community study from the ZOE COVID Study

Cristina Menni[1], PhD, Anna May[2], MA, Lorenzo Polidori[2], MSc, Panayiotis Louca[1], MSc, Jonathan Wolf[2], MA, Joan Capdevila[2], PhD, Christina Hu[2], MA, Prof Sebastien Ourselin[3],PhD, Claire J Steves[1], PhD, Prof Ana M. Valdes[1,4,*], PhD, Prof Tim D. Spector[1*], MD

APPENDIX

Table of contents

Supplementary Methods 2

Data sources 2

Ascertainment of covariates 2

Estimating vaccine effectiveness 2

Booster effectiveness 3

Secondary Analyses on Side Effects after the Booster Dose 3

Supplementary References 5

Supplementary Figure 1. Consort diagram 6

Supplementary Table 1. Outcomes for the vaccine effectiveness analysis across vaccine types and months since vaccination. 7

Supplementary Table 2. Sensitivity analysis of vaccine effectiveness at 5 months. VE in healthcare professionals, against symptomatic adjusted for covariates. 9

Supplementary Table 3. Descriptive characteristics of the ZOE Covid Study participants who were lost to follow-up. 10

Supplementary Table 4. Vaccine effectiveness against severe illness and hospitalisation analysis across vaccine types. 11

Supplementary Table 5. Vaccine effectiveness against severe illness and hospitalisation analysis across vaccine types, stratified by age group adjusted for covariates. 12

Supplementary Table 6. Outcomes for the vaccine effectiveness analysis across vaccine types for second dose against unvaccinated controls and third dose against second dose. 13

Supplementary Table 7. Descriptive characteristics of the study population that received the booster shot for the population of systemic/local side effects analysis after third doses, stratified by brand. 14

Supplementary Table 8. Occurrence rate of systemic and local side effects across different vaccination schedules adjusted for age, sex, BMI, healthcare worker status and presence of comorbidities. 16

Supplementary Methods

Data sources

The app was developed by the health data company Zoe Ltd with input from King's College London, the Massachusetts General Hospital, Lund University, and Uppsala University. It was launched on March 24, 2020 and freely available to download in the UK, as previously described [1]. App use was driven by referrals, word of mouth, the media and eventually partnerships with charities and the Welsh and Scottish Governments [2]. Adult contributors can also proxy-report for others, including children. Through direct updates to the application, new or modified questions are added in real time to capture data to test emerging hypotheses about COVID-19 symptoms and treatments.

Here we analysed data from the November 23rd, 2021 data dump, collected on versions 1.2.0, 1.4.1, 1.5.0, 1.5.1 and 1.6 of the app.

Ascertainment of covariates

Covariates including age, sex, BMI, smoking, race/ethnicity, healthcare worker status, and presence of comorbidities (i.e. cancer, diabetes, eczema, heart disease, lung disease, kidney disease and hay fever) were self-reported via the app. We also considered previous infection (binary variable) and number of tests as potential confounders. Finally, we estimated weekly incidence pe[3]r million in the UK at the time of the infection as previously described [3].

Estimating vaccine effectiveness

We compared the PCR/lateral flow test outcomes of doubly vaccinated individuals with those of unvaccinated users reporting a COVID-19 test on the same day. We computed the difference in months between when participants had their second vaccine dose and when they were tested, and we used this metric to group users. For each of the vaccines and for different time points from the second vaccination date, we used Poisson regressions to model the rates of positive tests in doubly vaccinated individuals compared to the unvaccinated controls, adjusting for age (≤ 55 and > 55 years), sex, previous infection (binary variable), healthcare worker status (binary variable), comorbidities (binary variable, with or without comorbidities), number of tests and weekly incidence per million in the UK at the time of the infection to control for the background positivity level as previously described[4].

We further tested the role of covariates on risk of infection post-vaccination by running stratified Poisson models (adjusted for confounders as above) on categories of age and comorbidities. For this analysis, we considered all app responders who were vaccinated with the second dose of BNT162b2 or ChAdOx1 nCoV-19 vaccine at least 14 days before having a test for SARS-CoV-2 positivity

Booster effectiveness

This analysis was only carried out on individuals aged 55 or older (both those with booster and without) because by November 23rd, 2021 most individuals under 55 were not eligible to receive a booster, hence it was not possible to establish a control group for younger individuals who had received the booster. We included post-booster PCR or lateral flow test results beyond the first 14

days and before 3 months after the booster vaccination date for the treatment group and post-second dose test results beyond the first 14 days and before 3 months after the second dose vaccination date for the control group. The 3 month limit corresponds to the end of follow-up given the period of observation included in our study for the booster. We used the same adjusted Poisson regressions as above to compare the PCR/lateral flow test outcomes of individuals who had been vaccinated with a booster with those of second dose vaccinated individuals reporting a COVID-19 test. We obtained the estimate of the log difference in the positivity rates of the booster vaccinated individuals and second dose vaccinated controls from the Poisson regression model. We combined the estimated difference between booster and second dose vaccinated individuals to the estimated risk reduction compared to unvaccinated individuals (measured at 0-3 months post vaccination). This allowed us to calculate an estimate of the vaccine effectiveness post booster compared to unvaccinated controls as follows:

$$VE^{booster\ vs\ unvaccinated} = 1 - RR^{booster\ vs\ 2nd\ dose}{}_{j,0\text{-}3} * RR^{2nd\ dose\ vs\ unvaccinated}{}_{,0\text{-}3}$$

where $RR^{booster\ vs\ 2nd\ dose}{}_{j,0\text{-}3}$ is the risk ratio coming from the booster effectiveness model *and* $RR^{2nd\ dose\ vs\ unvaccinated}{}_{,0\text{-}3}$ is the risk ratio from the 2nd dose effectiveness model, both measured at 0 - 3 months post vaccination for i,j ϵ [BNT162b1, ChAdOx1 nCoV-19 and mRNA-1273].

Secondary Analyses on Side Effects after the Booster Dose

For this secondary analysis, we included fully vaccinated individuals receiving a booster dose and logging their systemic and/or local effects (or the absence of those) at least once within 8 days from the vaccination date.

We estimated the ratio of the daily number of users reporting at least one adverse effect (systemic or local) after vaccination over the total number of vaccinated users logging in that day. As booster shots in the UK are performed with either BNT162b2 or mRNA-1273[5] and as the number of people who received a homologous booster with mRNA-1273 is limited because of vaccine roll out, we compared the probability of having adverse effects following homologous (BNT162b2) or heterologous (ChAdOx1 nCoV-19- BNT162b2; BNT162b2 -mRNA-1273; ChAdOx1 nCoV-19 -mRNA-1273) shots.

As described above, we compute the reactogenicity of different vaccines using Pearl's adjustment formula

$$P(R|do[V]) = \sum^{S} P(R|S,V)P(S)$$

where *R* is adverse effects, *S* is the set of confounder variables (i.e. age (<=55 years *vs* >55 years), in line with stratification in the BNT162b2 and ChAdOx1 nCoV-19 phase 3 trials, sex, health-care worker status (binary variable), obesity (BMI <30 kg/m^2 *vs* ≥30 kg/m^2), and comorbidities (binary variable, with or without comorbidities), and $P(R \mid S, V)$ is the probability of having adverse effects in a given stratum after receiving a vaccine V ϵ [BNT162b1, ChAdOx1 nCoV-19 and mRNA-1273]. The odds ratios when comparing reactogenicity across vaccines are computed as:

$$OR = \frac{\frac{P(R|do[V_1])}{1 - P(R|do[V_1])}}{\frac{P(R|do[V_2])}{1 - P(R|do[V_2])}}$$

where $P(R|do[V_j])$is the probability of having side effects R given a specific treatment V_j.

Supplementary References

1 Drew DA, Nguyen LH, Steves CJ, *et al.* Rapid implementation of mobile technology for real-time epidemiology of COVID-19. *Science* 2020; **368**: 1362–7.

2 Menni C, Valdes AM, Freidin MB, *et al.* Real-time tracking of self-reported symptoms to predict potential COVID-19. *Nat Med* 2020; **26**: 1037–40.

3 Varsavsky T, Graham MS, Canas LS, *et al.* Detecting COVID-19 infection hotspots in England using large-scale self-reported data from a mobile application: a prospective, observational study. *Lancet Public Health* 2021; **6**: e21–9.

4 Menni C, Klaser K, May A, *et al.* Vaccine side-effects and SARS-CoV-2 infection after vaccination in users of the COVID Symptom Study app in the UK: a prospective observational study. *Lancet Infect Dis* 2021; **21**: 939–49.

5 COVID-19 vaccines. https://vk.ovg.ox.ac.uk/vk/covid-19-vaccines (accessed Dec 4, 2021).

Supplementary Figure 1. Consort diagram

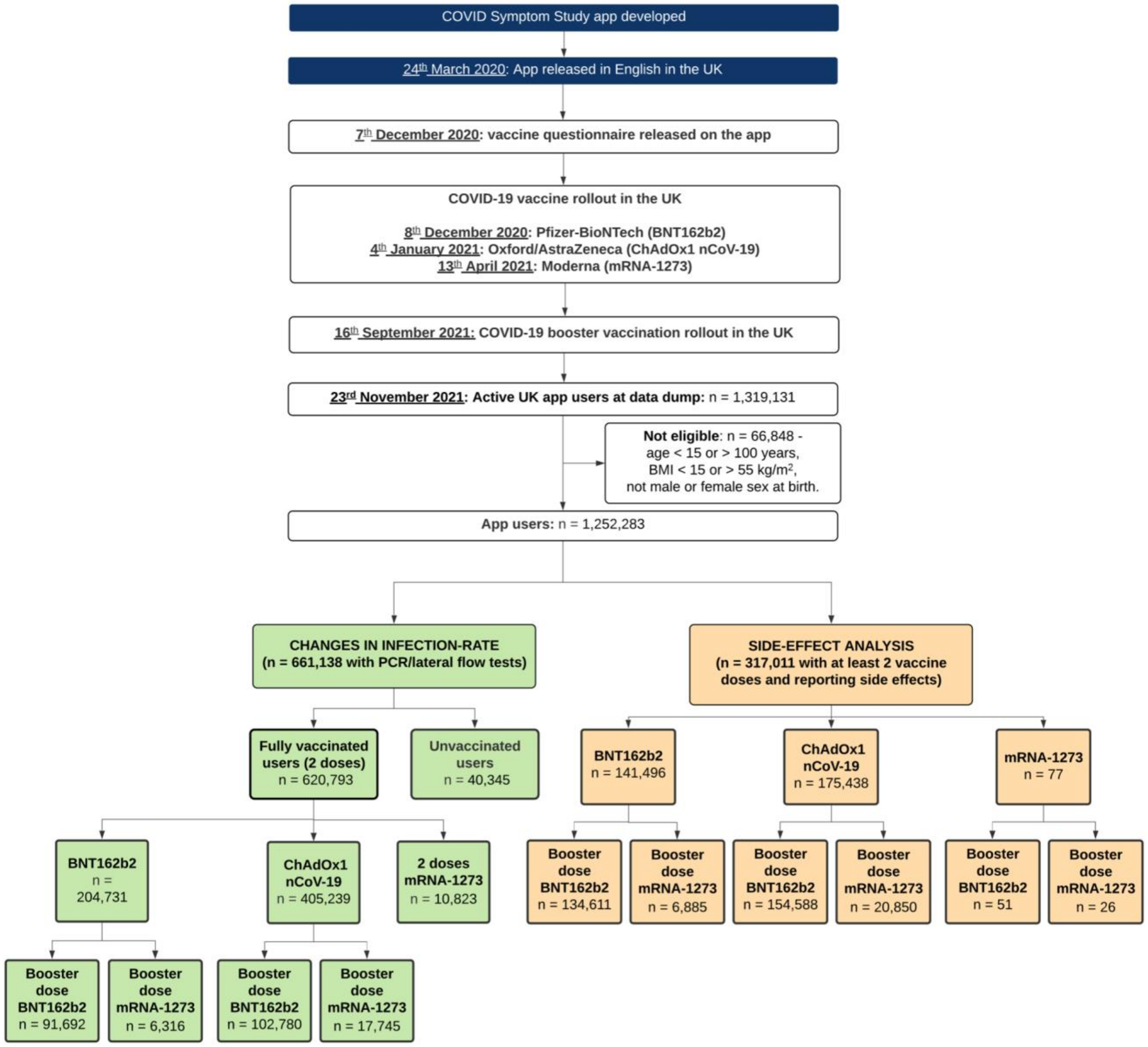

Supplementary Table 1. Outcomes for the vaccine effectiveness analysis across vaccine types and months since vaccination.

Unvaccinated controls are employed to compute raw vaccine effectiveness; the adjusted rate is also included.

Vaccine type	Months since vaccination	Positive tests (control = 6701)	Number of tests (control = 90157)	Raw vaccine effectiveness	Adj vaccine effectiveness
**ChadOx1 nCoV-19**	1	1682	198419	88.6	83.1% [82.2 - 84]
	2	5759	444233	82.6	79.3% [78.6 - 80.0]
	3	7952	464711	77.0	76.7% [76.0 - 77.5]
	4	9669	482535	73	75.8% [75.0 - 76.4]
	5	11067	467213	68.1	75.7% [74.9 - 76.4]
	6	9067	370328	67.1	75.2% [74.3 - 76.1]
**BNT162b2**	1	407	60066	90.9	91.6% [90.7 - 92.4]
	2	1230	164555	89.9	89.0% [88.3 - 89.7]

	3	2291	231587	86.7	86.8%<br>[86.2 - 87.4]
	4	3233	236283	81.6	83.7%<br>[82.9 - 84.3]
	5	2973	213924	81.3	82.1%<br>[81.3 - 82.9]
	6	3231	205246	78.8	81.6%<br>[80.8 - 82.4]
	7	2065	105039	73.5	79.4%<br>[78.3 - 80.5]
	8	555	22645	67.0	75.7%<br>[73.4 - 77.7]
**mRNA-1273**	1	54	7872	90.8	94.1%<br>[92.3 - 95.5]
	2	135	13844	86.9	91.7%<br>[90.2 - 93.0]
	3	194	13742	81.0	89.9%<br>[88.4 - 91.3]
	4	236	11067	71.3	87.7%<br>[86.0 - 89.2]
	5	121	4727	65.6	84.3%<br>[81.2 - 86.9]

Supplementary Table 2. Sensitivity analysis of vaccine effectiveness at 5 months. VE in healthcare professionals, against symptomatic adjusted for covariates.

Vaccine Type	Months since vaccinated	VE for healthcare workers only	VE for those not previously infected	VE for symptomatic positives*
ChAdOx1 nCoV-19	5	73.4% [68.9-77.3]	75.8% [75.0-76.6]	72.6% [71.5-73.6]
BNT162b2	5	80.2% [77.5-82.6]	82.2% [81.4-83]	80.9% [79.9-81.8]
mRNA-1273	5	*cannot be estimated*	84.7% [81.6-87.2]	83.0% [79.4-85.9]

* VE for symptomatic positives is measured for all individuals who filled a symptoms assessment and who reported symptoms of Covid-19 (see Table 1, also for number of healthcare workers).

Supplementary Table 3. Descriptive characteristics of the ZOE COVID Study participants who were lost to follow-up. Data are n(%) unless otherwise indicated

	**BNT162b2**<br>**(N=71,528)**	**ChAdOx1 nCoV-19**<br>**(N=146,088)**	**mRNA-1273**<br>**(N=4,017)**
Sex			
Female	42,969(60.1%)	83,289(57.0%)	2,218(55.2%)
Male	28,559(39.9%)	62,799(43.0%)	1,799(44.8%)
Age, years	52.2(18.8); median: 52 (IQR: 36-67)	55.9(12); median: 56 (IQR: 49-64)	38.6(9.9); median: 39 (IQR: 32-46)
BMI, kg/m^2	26.6(5.6)	27.0(5.5)	25.6(5.0)
Health-care workers	5,769(8.1%)	2,730(1.9%)	53(1.3%)
Comorbidities	15,118(21.1%)	24,858(17.0%)	306(7.6%)

Supplementary Table 4. Vaccine effectiveness against severe illness and hospitalisation analysis across vaccine types. Unvaccinated controls are employed to compute raw vaccine effectiveness, the adjusted rate is also included (adjusted for age (≤ 55 and > 55 years), sex, previous infection (binary variable), healthcare worker status (binary variable), comorbidities (binary variable, with or without comorbidities), number of tests and weekly incidence per million in the UK at the time of the infection to control for the background positivity level).

Vaccine type	Months since vaccination	Positive tests with severe symptoms (control = 986)	Number of tests (control = 77791)	Raw vaccine effectiveness against severe infection	Adjusted vaccine effectiveness against severe infection
Any vaccine *	5-6	4421	1242243	71.9%	78.8% [77.1-80.3]
ChAdOx1 nCoV-19	5-6	3467	826419	66.9%	75.45%[73.5-77.3]
BNT162b2	5-6	944	411144	81.9%	85.1%[83.6-86.4]

Vaccine type	Months since vaccination	Positive tests with hospitalisation (control = 186)	Number of tests (control = 77791)	Raw vaccine effectiveness against hospitalisation	Adjusted vaccine effectiveness against hospitalisation
Any vaccine*	5-6	685	1242243	76.9%	84.1% [81.0-86.7]
ChAdOx1 nCoV-19	5-6	549	826419	72.2%	81.0%[77.1-84.2]
BNT162b2	5-6	134	411144	86.4%	89.6%[86.9-91.8]

*including mRNA-1273

Supplementary Table 5. Vaccine effectiveness against severe illness and hospitalisation analysis across vaccine types, stratified by age group adjusted for covariates.

Vaccine type	**VE against severe illness**<br>*age <55 years*	**VE against severe illness**<br>*age >=55 years*	**VE against hospitalisation**<br>*age <55 years*	**VE against hospitalisation**<br>*age >=55 years*
Any vaccine*	79.2% [77.4-80.8]	66.5% [57.5-73.5]	84.3% [80.7-87.2]	80.4% [70.7-86.9]
ChAdOx1 nCoV-19	76.4% [74.3-78.4]	61.0% [50.4-69.3]	81.0% [76.3-84.7]	77.5% [66.1-85]
BNT162b2	85.4% [83.8-86.8]	76.9% [70.1-82.2]	90.7% [87.5-93.0]	86.0% [77.8-91.1]

*including mRNA-1273

Supplementary Table 6. Outcomes for the vaccine effectiveness analysis across vaccine types for second dose against unvaccinated controls and third dose against second dose. Unvaccinated controls and double vaccinated controls are employed to compute raw vaccine effectiveness; the combined rate for vaccine effectiveness of third doses against unvaccinated controls is also included. As described in page 3 and 4, adjusted VE is computed by taking

$$VE_{\text{booster vs unvaccinated}} = 1 - RR_{\text{booster vs 2nd dosej,0-3}} * RR_{\text{2nd dose vs unvaccinated,0-3}}$$

2nd dose vaccine	Booster vaccine	Positive tests (unvaccinated control = 730)	Number of tests (unvaccinated control = 20779)	Raw RR (second dose vs unvaccinated)	Adjusted RR (second dose vs unvaccinated)	
ChadOx1 nCoV-19	None	4518	592947	0.3	0.3 [0.3-0.3]	
BNT162b2	None	578	186653	0.3	0.1 [0.2-0.1]	
**2nd dose vaccine**	**Booster vaccine**	**Positive tests (ChadOx1 nCoV-19 control = 796, BNT162b2 control = 28)**	**Number of tests (ChadOx1 nCoV-19 control = 48013, BNT162b2 control = 2046)**	**Raw RR (third dose vs second dose)**	**Adjusted RR (third dose vs second dose)**	**Adjusted VE (third dose vs unvaccinated)**
ChadOx1 nCoV-19	mRNA-1273	64	6891	0.2	0.4 [0.5-0.3]	88.8% [84.4-92.0]
ChadOx1 nCoV-19	BNT162b2	965	116777	0.2	0.3 [0.3-0.3]	91.0% [89.2-92.5]
BNT162b2	BNT162b2	948	162529	0.2	0.3 [0.5-0.2]	95.3% [92.3-97.1]
BNT162b2	mRNA-1273	29	3437	0.2	0.5 [0.9-0.3]	92.5% [86-96]

Supplementary Table 7. Descriptive characteristics of the study population that received the booster shot for the population of systemic/local side effects analysis after third doses, stratified by brand. Data are n(%) unless otherwise indicated

	Booster doses		
	**Overall (N = 317,011)**	**BNT162b2 (N = 289,250)**	**mRNA-1273 (N = 27,761)**
Sex			
Female	184,497 (58.2%)	168,642 (58.3%)	158,55 (57.1%)
Male	132,514 (41.8%)	120,608 (41.7%)	119,06 (42.9%)
Homologous dose	134,637 (42.5)	134,611 (46.5%)	26 (0.09%)
Age, years (mean(SD))	65.4 (10.6)	65.6 (10.7)	62.8 (8.4)
BMI, kg/m^2	26.5 (5.1)	26.5 (5.1)	26.7 (5.2)
Healthcare workers	18025 (5.7%)	17499 (6.0%)	526 (1.9%)
Comorbidities	82659(26.1%)	76818 (26.6%)	6414 (21.0%)
**Systemic side-effects**			
Any	50,339 (15.9%)	43,925 (15.2%)	7,391 (22.9%)
Headache	28,563 (9.0%)	24,765 (8.6%)	3,798 (13.7%)
Fatigue	31,881 (10.1%)	27,703 (9.6%)	4,178 (15.0%)
Chills and Shivers	13,648 (4.3%)	11,329 (3.9%)	2,319 (8.4%)
Diarrhoea	3,884 (1.2%)	3,523 (1.2%)	361 (1.3%)
Fever	7,486 (2.4%)	6,301 (2.2%)	1,185 (4.3%)
Arthralgia	14,189 (4.5%)	12,129(4.2%)	2,060 (7.4%)
Myalgia	8,480 (2.7%)	7,267 (2.5%)	1,213 (4.4%)
Nausea	6,614 (2.1%)	5,691 (2.0%)	9,23 (3.3%)
**Local side-effects**			
Any	232,596 (73.4%)	209,954 (72.6%)	22,642 (81.6%)
Pain	92,570 (29.2%)	81,566 (28.2%)	11,004 (39.6%)
Swelling	25,154 (4.7%)	21,530 (7.4%)	3,624 (13.1%)
Tenderness	187,767 (59.2%)	169,296 (58.5%)	18,471 (66.5%)
Itch	9,983 (3.1%)	88,76 (3.1%)	1,107 (4.0%)

Swollen armpit glands	8,321 (2.6%)	7,549 (2.6%)	772 (2.8%)
Redness	14,908 (4.7%)	13,000 (4.5%)	1,908 (6.9%)
Warmth	25,829 (8.1%)	22574 (7.8%)	3,255 (11.7%)
Bruising	13845(4.4%)	12,240 (4.2%)	1,605 (5.8%)

Data are n, n (%), mean (SD) for age and BMI.. BMI=body-mass index.

Supplementary Table 8. Occurrence rate of systemic and local side effects across different vaccination schedules adjusted for age, sex, BMI, healthcare worker status and presence of comorbidities.

Primary vaccination	Booster dose	n/N systemic	adjusted % of systemic effects	n/N local	adjusted % of localised effects
ChadOx1 nCoV-19	BNT162b2	41,918/154,588	16.1% [15.9 - 16.2]	125,442/154,588	61.9% [61.7 - 62.1]
BNT162b2	mRNA-1273	2,207/6,885	18% [17.1- 18.8%]	6,296/6,885	79.2% [78.4 - 80.0]
BNT162b2	BNT162b2	17,698/134,611	13.2% [13.0 - 13.3]	95,901/134,611	71.2% [71.0 - 71.5]
